# Genomic and biochemical approaches in the discovery of mechanisms for selective neuronal vulnerability to oxidative stress

**DOI:** 10.1186/1471-2202-10-12

**Published:** 2009-02-19

**Authors:** Xinkun Wang, Asma Zaidi, Ranu Pal, Alexander S Garrett, Rogelio Braceras, Xue-wen Chen, Mary L Michaelis, Elias K Michaelis

**Affiliations:** 1 Higuchi Biosciences Center, 2099 Constant Ave., University of Kansas, Lawrence, KS 66047, USA; 2 Department of Pharmacology and Toxicology, 1251 Wescoe Dr., University of Kansas, Lawrence, KS 66045, USA; 3 Current address: Department of Biochemistry, 1750 Independence Ave., Kansas City University of Medicine and Biosciences, Kansas City, MO 64106, USA; 4 Bioinformatics and Computational Life Sciences Laboratory, Department of Electrical Engineering and Computer Science, 2335 Irving Hill Rd., University of Kansas, Lawrence, KS 66045, USA; 5 Current address: Stowers Institute for Medical Research, 1000 E 50th St., Kansas City, MO 64110, USA; 6 Current address: Daiichi Sankyo Inc., Two Hilton Court, Parsippany, NJ 07054, USA

## Abstract

**Background:**

Oxidative stress (OS) is an important factor in brain aging and neurodegenerative diseases. Certain neurons in different brain regions exhibit selective vulnerability to OS. Currently little is known about the underlying mechanisms of this selective neuronal vulnerability. The purpose of this study was to identify endogenous factors that predispose vulnerable neurons to OS by employing genomic and biochemical approaches.

**Results:**

In this report, using *in vitro *neuronal cultures, *ex vivo *organotypic brain slice cultures and acute brain slice preparations, we established that cerebellar granule (CbG) and hippocampal CA1 neurons were significantly more sensitive to OS (induced by paraquat) than cerebral cortical and hippocampal CA3 neurons. To probe for intrinsic differences between *in vivo *vulnerable (CA1 and CbG) and resistant (CA3 and cerebral cortex) neurons under basal conditions, these neurons were collected by laser capture microdissection from freshly excised brain sections (no OS treatment), and then subjected to oligonucleotide microarray analysis. GeneChip-based transcriptomic analyses revealed that vulnerable neurons had higher expression of genes related to stress and immune response, and lower expression of energy generation and signal transduction genes in comparison with resistant neurons. Subsequent targeted biochemical analyses confirmed the lower energy levels (in the form of ATP) in primary CbG neurons compared with cortical neurons.

**Conclusion:**

Low energy reserves and high intrinsic stress levels are two underlying factors for neuronal selective vulnerability to OS. These mechanisms can be targeted in the future for the protection of vulnerable neurons.

## Background

Oxidative stress (OS) is an important factor in brain aging and some neurodegenerative diseases [1-4]. Under normal conditions, the processes of generating and scavenging reactive oxygen (ROS) and nitrogen species (RNS) are in equilibrium. Excessive production of ROS or RNS leads to oxidative modification and altered functional states of proteins, nucleic acids, and lipids. During aging and in certain diseased states, this equilibrium is disrupted and selectively affects neuronal survival in specific brain regions. The selective effects of OS on neurons are manifested as cell death in restricted populations of neurons while many other neurons appear to cope with the stress induced by excess ROS or RNS production [5-8]. Selective neuronal vulnerability (SNV), such as that seen following OS, has also been observed following other brain insults, for example, glutamate excitotoxicity, ischemia, or β-amyloid-induced neurotoxicity [9-13]. In order to shed more light on SNV in general, transcriptomic analyses of neurons that exhibit differential vulnerability to various insults or to the damage brought about by neurological diseases have been performed in human and rodent hippocampus and human midbrain dopaminergic neurons [14-19]. However, none of these studies except for the one on dopaminergic neurons focused on a specific form of stress, or on genes or bio-functions that might contribute to the etiology of SNV.

It is important to note that a common pathway to neuronal injury resulting from the various forms of brain insult mentioned above is believed to be that of induction of intracellular OS. Yet, there is currently little information on the mechanisms for SNV to OS. Since OS-sensitive neurons might be the ones that degenerate early during the aging process or in certain neurodegenerative diseases [1], study of the molecular mechanisms of SNV to OS may offer insights into both aging-associated and disease-initiated neurodegeneration, as well as provide leads to the protection of vulnerable neurons. To study the relationship between SNV and OS, we thought it necessary to determine differences in the redox status and OS-handling capacity of both OS-sensitive and OS-resistant neurons. In a previous study, we found molecular indications of an intrinsically high level of oxidative activity under baseline conditions in OS-vulnerable CA1 when compared with OS-resistant CA3 neurons in organotypic cultures maintained *in vitro *[20]. In a subsequent study, we examined how neurons in CA1 and CA3 responded differentially to OS increases in terms of the neuronal gene expression patterns and we identified genes whose expression distinguished the responses of CA1 from those of CA3 neurons [21]. Since our previous studies were performed on neurons maintained *in vitro *in organotypic cultures, the patterns of gene expression might not have been identical to those of neurons in the intact brain *in vivo*. Furthermore, in order to advance our understanding of mechanisms of SNV to OS, it was considered important to probe for differences between vulnerable and resistant neurons extracted from several brain regions besides the hippocampus pyramidal neuron layers, and to do so with neurons in their native states.

The identification and inclusion of more than two neuronal populations that are either susceptible or resistant to OS should help in revealing more generalized patterns of gene expression associated with SNV. This was thought to be the case as some of the results obtained from comparative analyses of only CA1 *vs*. CA3 may simply reflect regional or ontogenetic differences between these two neuronal populations rather than selective vulnerabilities to stressors like OS. In initial studies described in this paper, we found that cerebellar granule (CbG) cells were a group of cells that were quite vulnerable to OS, whereas neurons from the cerebral cortex were relatively resistant. Based on these observations, we included both cortical and CbG neurons to our genomic and biochemical investigations of SNV. In order to identify endogenous predisposing factors that may lead to the selective vulnerability of CA1 and CbG neurons to OS, we employed laser capture microdissection (LCM) to collect these target neurons from freshly prepared brain sections under basal conditions (no OS induction), and compared their transcriptomic profile with that of the CA3 and cortex neurons. Following the findings obtained from the functional genomics study, targeted biochemical analyses on key antioxidant enzymes and ATP generation were carried out.

## Results

### CbG as well as CA1 neurons exhibit vulnerability to OS

**Figure 1:**
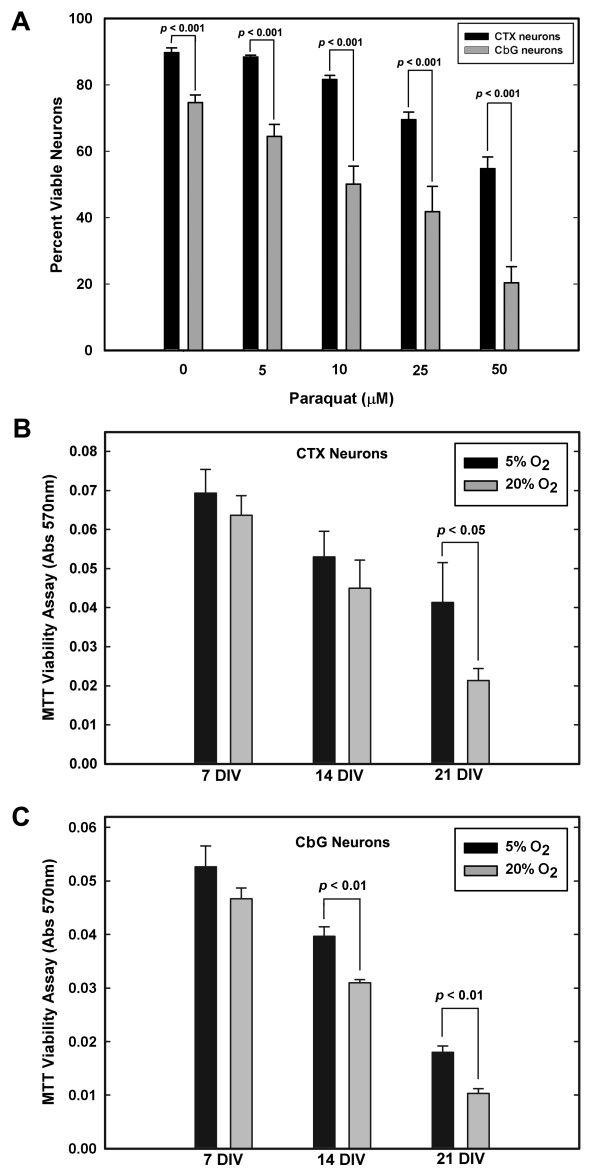
**Effect of paraquat and of differential levels of oxygen tension on the viability of primary CbG and cerebral cortical (CTX) neurons**. (A) Effects of paraquat on neuronal viability. Both types of neurons were exposed to the indicated concentrations of paraquat for 24 h at 37°C. Cell viability was determined by the calcein AM and PI labeling method. Data represent the mean (± SEM) of neuronal viability from 10 experiments. The *P *values from Student's *t*-test are shown for the indicated comparisons. (B, C) Survival of neurons across days *in vitro *(DIV) under different levels of oxygen (5% and 20%). Viability of neurons was measured by the MTT assay. The data represent the mean (± SEM) from 3 experiments. Statistically significant differences (*t*-test) are shown for the indicated comparisons.

We first attempted to determine if primary neuronal cultures of CbG and cerebral cortical cells were differentially sensitive to OS. Despite the caveat that studies performed with neurons grown *in vitro *may not reflect exactly the responses of neurons *in vivo*, primary neuronal cultures were used in these studies because they are composed of approximately 90–95% neurons. Thus, differential responses to OS could be directly attributed to specific characteristics of neurons from these two brain regions. Oxidative stress was induced by exposing the neuronal cultures to various concentrations of paraquat (0–50 μM). Paraquat induces OS by generating superoxide in neurons through the process of one-electron reduction by the flavoenzyme NADPH-cytochrome P450 reductase [22-25]. Increases in paraquat concentration led to a dose-dependent increase in cell death in both types of neurons, but the effect of paraquat on neuronal survival was greater for CbG than cerebral cortical neurons. For example, treatment with the highest concentration of paraquat (50 μM) caused approximately 80% neuronal death in CbG but 40% in cerebral cortical neurons (Fig. 1A). Two-way ANOVA indicated that the effect of paraquat treatment on survival in the two types of cells was statistically significant (*df *= 4, *F *= 42.822, *P *< 0.001). The viability of CbG neurons was significantly lower than that of cortical neurons across all paraquat concentrations (*t*-test, *P *values < 0.001).

Since oxygen (O_2_) tension in the ambient air surrounding the medium of primary neuronal cultures is ~20% (by volume), i.e., higher than that in the body, an important difference between neurons in culture as compared with those in the intact brain may be the fact that primary neurons in culture are continuously exposed to high O_2 _concentrations and, possibly, some level of OS. Thus, the effects of paraquat on cell viability of primary neurons might have been a combined effect of exposure of these neurons to increased O_2 _tension and to paraquat. In order to determine whether different levels of O_2 _tension, in the absence of an ROS-generating chemical agent, were associated with differential neuronal viability, we compared neuronal survival over 21 days *in vitro *(DIV) for neurons in cultures maintained under either reduced (5%) or high O_2 _(20%) atmosphere. While high O_2 _tension had no significant effect on the survival of cerebral cortical neurons grown in culture for 7 and 14 DIV when compared with survival in low O_2 _tension, the survival of cortical neurons grown for 21 days under reduced O_2 _tension (5% O_2_) exhibited significantly higher survival than neurons grown for 21 DIV under high O_2 _(20% O_2_) tension (Fig. 1B). The effect of high O_2 _tension on the survival of CbG neurons was greater than that on cerebral cortical neurons. CbG neurons grown for either 14 or 21 DIV under 5% O_2 _tension had significantly higher survival than those grown under 20% O_2 _tension (Fig. 1C). Two-way ANOVA testing the effects of neuronal type and O_2 _tension on cell viability showed that, overall, cortical neurons had significantly higher viability than CbG neurons (*df *= 1, *F *= 8.646, *P *= 0.006). Three-way ANOVA testing time of exposure, O_2 _tension, and cell type showed significant interactions (all *P *values < 0.001). *Post-hoc *analyses using the Holm-Sidak method indicated significant effects of time of exposure (*df *= 2, *F *= 130.825, *P *< 0.001), O_2 _tension (*df *= 1, *F *= 27.296, *P *< 0.001), and cell type (*df *= 1, *F *= 79.106, *P *< 0.001) on cell viability. These data demonstrated that, even in the absence of agents that generate intracellular ROS, CbG neurons were more sensitive to changes in environmental O_2 _tension than cortical neurons.

The conditions of primary cultures of cerebral cortical neurons differed from those used in primary cultures of CbG neurons and may thus have affected the overall response of these two types of neurons to paraquat-induced OS. In order to determine the effects of OS under uniform conditions of cell growth and maintenance *in vitro*, we also examined the effects of paraquat on neuronal viability in organotypic slice cultures of cerebral cortex and cerebellum. The conditions of growth of slice cultures were the same for both cortical and cerebellar cultures. Organotypic slice cultures would also allow us to determine if the differential vulnerability of CbG and cortical neurons observed in primary neuronal cultures would be observed in brain sections that contain both neurons and glia. Organotypic slice cultures maintain a relatively normal brain cyto-architecture while cells are growing in culture. Such cultures were previously used by us and others to document the differential vulnerability to OS of hippocampus neurons of the CA1 and CA3 regions [5,20]. In these previous studies, the superoxide-generating agent employed was duroquinone. But, in the present studies, the investigations were expanded to include a different agent whose metabolism leads to superoxide formation, paraquat, and thus assess whether the cell death produced was related to the common property of these two chemical agents, i.e., superoxide generation in cells.

**Figure 2:**
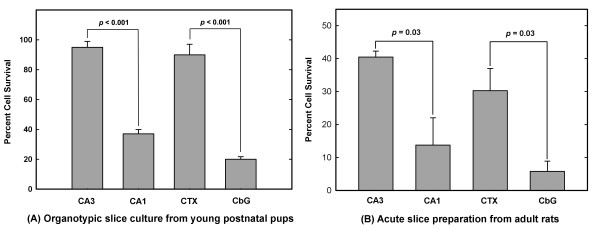
**Selective vulnerability of hippocampal CA1 and CbG neurons as compared with CA3 and CTX neurons in organotypic brain slice cultures and in acute slice preparations**. Both organotypic brain slice cultures and acute slice preparations were treated with paraquat (100 μM, 1 h). Cell survival was determined by PI labeling. Data represent the mean ± SEM. The number of experiments were n = 6 for (A) and n = 3 for (B). Statistically significant differences (*t*-test) are shown for the indicated comparisons.

In the present study, we employed organotypic slice cultures of cerebral cortex, cerebellar cortex, and hippocampus, including the CA1 and CA3 regions, all grown and exposed to either paraquat or vehicle under identical conditions. But, since the intent was to obtain neurons from freshly prepared brain slices for the purpose of transcriptomic analyses, i.e., fresh tissue at near *in vivo *conditions, we also examined the effects of paraquat on neuronal viability in hippocampus, cerebral and cerebellar cortex in acute brain slice preparations. The acute slice preparations were obtained from adult rats whereas those maintained *in vitro *for prolonged periods were obtained from 6–7 day-old pups. In the slice cultures maintained under prolonged incubation conditions *in vitro*, paraquat (final concentration 100 μM) was applied to the brain slices after they were in culture for one week and the effects on neuronal survival assessed 24 h after exposure to paraquat or vehicle. In the case of acutely prepared slices, the slices were incubated in culture medium for only a period of two hours before the same paraquat treatment. Although the cell survival rate was lower, in general, in cultures established from adult animals in comparison to those from young pups, greater neuronal death following paraquat exposure was observed in the CA1 and CbG regions as compared with the CA3 and cerebral cortex, and that was the case in both prolonged organotypic slice cultures and acute slice preparations (Fig. 2). Thus, in terms of sensitivity to OS, CbG neurons were similar to CA1 hippocampus neurons, whereas cortical neurons were similar to CA3 neurons. The presence of glial cells in slice cultures did not seem to alter the relative vulnerability of CbG and cortical neurons to OS.

### Comparative biochemical analyses of CbG and cerebral cortical neurons

**Figure 3:**
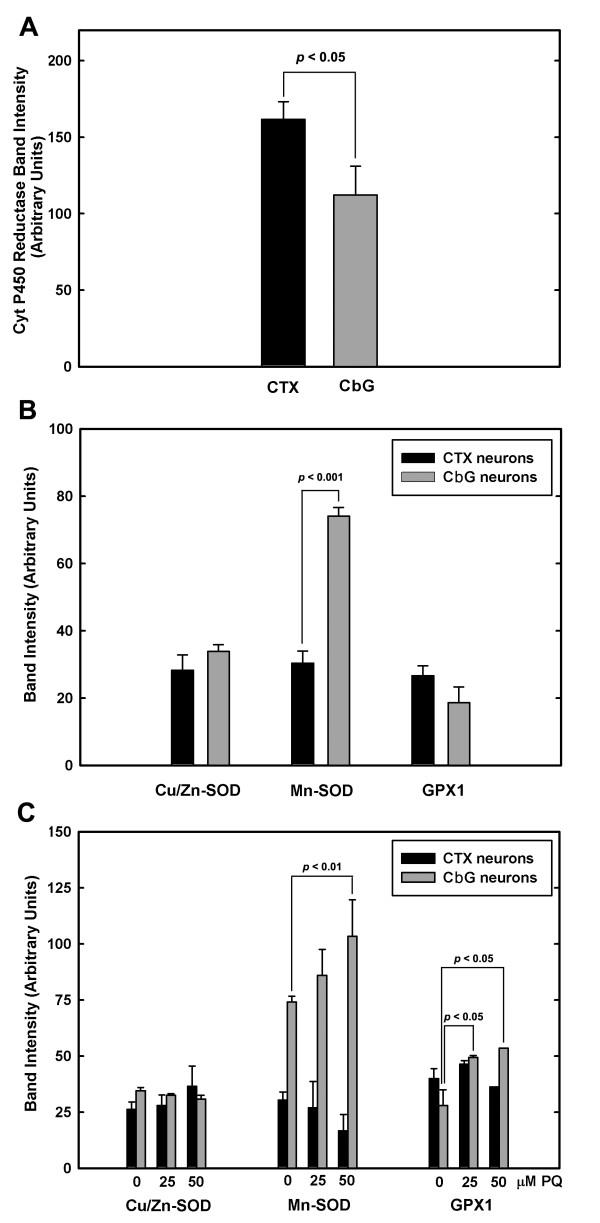
**Protein levels of NADPH-cytochrome P450 reductase and for cellular oxidoreductases in CTX and CbG neurons**. (A) Protein levels of NADPH-cytochrome P450 reductase in neurons. Immune labeling with anti-cytochrome P450 reductase antibodies was performed on proteins extracted from neurons cultured under conditions of 20% O_2 _tension. Results of densitometric measurements of the immuno-reactive band in each immunoblot are shown as the mean (± SEM) of 5 experiments. Statistically significant differences (*t*-test) are shown for the indicated comparisons. (B) Protein levels of SOD1, SOD2, and GPX1 in neurons. Immune labeling with anti-SOD1, anti-SOD2, and anti-GPX1 antibodies was performed on proteins extracted from neurons cultured as described in A. Results of densitometric measurements of the immuno-reactive bands in each immunoblot are shown as the mean (± SEM) of 3 experiments. Statistically significant differences (*t*-test) are shown for the indicated comparisons. (C) Effect of paraquat-induced OS on the protein levels of SOD1, SOD2, and GPX1 in neurons. Primary neurons in culture were exposed to the indicated concentrations of paraquat for 24 h at 37°C. Immune labeling with antibodies was performed as described in B. Results of densitometric measurements of the immuno-reactive bands in each immunoblot are shown as the mean (± SEM) of 3–5 experiments. Statistically significant differences (*t*-test) are shown for the indicated comparisons.

The observed differential sensitivity of CbG and cortical neurons to paraquat and superoxide-induced OS, regardless of the culture type, culture conditions, or the relative chronicity of *in vitro *incubation of the cultures, afforded a good opportunity to conduct preliminary studies to determine whether differences in either the metabolism of paraquat or in the handling of superoxide by neurons might account for the differential vulnerabilities between these two neuronal populations. As mentioned above, paraquat generates superoxide anions through one-electron transfer reactions catalyzed by NADPH-cytochrome P450 reductase. Therefore, we first focused on potential differences in the levels of NADPH-cytochrome P450 reductase between CbG and cortical neurons. The protein levels of this enzyme were higher in cerebral cortical than CbG neurons (Fig. 3A). Based on these observations, it might have been expected that cortical neurons would be more susceptible to superoxide generation than CbG neurons. However, this was not the case. Thus, enhanced superoxide formation by cytochrome P450 reductase could not account for the greater sensitivity of CbG neurons to OS.

We next considered the possibility that the vulnerable neurons expressed lower levels of key anti-oxidant enzymes. Cu/Zn-SOD (SOD1), Mn-SOD (SOD2) and glutathione peroxidase (GPX1), were selected as oxidoreductases whose differential levels of expression might determine differential vulnerabilities to OS. The levels of SOD1 and GPX1 did not differ significantly between cerebral cortical and CbG neurons (Fig. 3B). However, the protein levels of SOD2 were 2.5 times higher in CbG than cortical neurons (Fig. 3B). Thus, based on endogenous SOD2 levels, it might have been predicted that CbG neurons would be more resistant than cortical neurons, which was not the case. It is possible that because these studies were performed using primary cultures maintained at 20% oxygen atmosphere, the elevated SOD2 levels in CbG neurons might have reflected an enhanced response of these neurons to OS induced that might have been induced by the high O_2 _tension present under the culture conditions used.

To determine if exposure of cerebral cortical and CbG neurons to OS that was produced through increased superoxide generation could affect the expression of the three oxidoreductases, the levels of SOD1, SOD2, and GPX1 were measured in neurons treated with paraquat (Fig. 3C). Statistical analyses showed that the levels of SOD1 were not significantly altered following treatment of either CbG or cortical neurons with paraquat. However, the levels of both SOD2 and GPX1 were significantly increased in CbG but not cortical neurons following exposure to paraquat. Results of two-way ANOVA indicated that SOD2 content was in general significantly higher in CbG *vs*. cerebral cortical neurons (*df *= 1, *F *= 113.958, *P *< 0.001). Therefore, the higher levels of SOD2 in CbG as compared with cortical neurons grown in primary cultures could reflect an adaptive response to exposure to elevated levels of O_2 _tension and the resulting OS.

Overall, these results indicated that the OS-vulnerable CbG neurons had the capacity to respond to increases in OS through the expression of higher SOD2 levels as compared with cortical neurons. Furthermore, CbG neurons showed much greater up-regulation of GPX1 than did cerebral cortical neurons following exposure to paraquat. This would suggest that CbG neurons would be equally or more protected from OS-induced cell death than cortical neurons. In conclusion, CbG neurons neither had reduced capacity to generate superoxide from paraquat nor reduced levels of anti-oxidant enzymes that protect neurons from OS. These relatively straightforward explanations of possible mechanisms of differential vulnerabilities of neurons to OS failed to reveal a pathway that might account for these differential properties of neurons. For this reason, the subsequent studies were focused on a more global approach to characterization of differentiating molecular pathways among the neurons derived from each of the four regions of rat brain that exhibited differential susceptibility to OS.

### Comparative transcriptome analyses of OS-vulnerable and OS-resistant neurons under basal conditions

**Figure 4:**
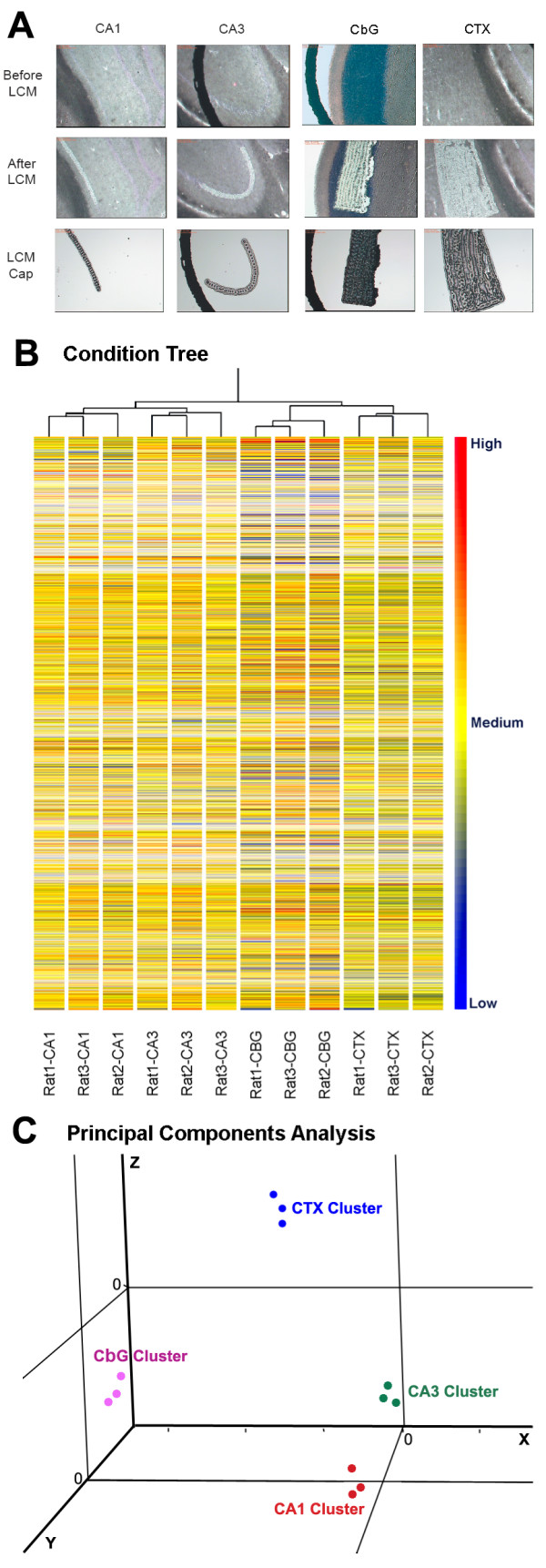
**LCM of neurons from the pyramidal cell layers of CA1 and CA3 of hippocampus, CbG cells of cerebellum, and layers IV-VI of cerebral cortex, and condition tree and Principal Components Analysis of microarray data from the captured neurons**. (A) LCM capture of neurons from the four brain regions. The upper row shows the lightly stained populations of neurons in the respective brain sections, the middle row shows the image of the sections after neuronal capture by LCM, and the bottom row shows the captured neurons on the LCM cap. (B) Condition tree analysis of microarray data. Condition tree analysis was based on expression values of all probesets on the Affymetrix RAE230A GeneChip (color bar shown at right indicates relative gene expression levels). Pearson correlation was used as measurement of similarity. (C) PCA of microarray data. This analysis allowed for the visualization of the relationships among samples from the four brain regions.

In order to obtain a global view of molecular processes that might predispose CA1 and CbG neurons to greater sensitivity to OS compared with CA3 and cerebral cortical neurons, we performed transcriptomic analyses of neurons from these four brain regions. These studies were performed under basal conditions, i.e., no paraquat-induced exogenous OS. In order to minimize non-target noise that might result from variations in sample collection and preparation, we employed LCM for the collection of highly homogeneous neuronal populations [26,27]. The homogeneity in the neuronal populations collected from the four designated brain regions is shown in the representative sections in Fig 4A. The cryosections used for LCM were prepared from rat brains frozen immediately after sacrifice, thus preserving, as much as possible, their native state in the brain and allowing us to assess the molecular differences among neurons under conditions that are likely to exist *in vivo*. The conditions for LCM dissection and recovery of neurons from the four brain regions were similar to those employed in the preparation of the acute adult brain slices that were used to detect the differential responses of neurons to paraquat-induced OS. Thus, uncovering differences under basal, near *in vivo *conditions, in the transcriptomic patterns between neurons that were vulnerable and those that were resistant to OS, might be indicative of the molecular pathways that are involved in determining the differential sensitivities to OS. Because the transcriptomic analyses planned were focused on differences detected under basal conditions, the rats used in the microarray studies were not subjected to any treatment that would induce OS.

Excellent reproducibility among replicate samples in GeneChip analysis was achieved. Non-target, systematic noise was very low. The mean correlation coefficient between replicate chips was 0.96 (range 0.91–0.99). Condition Tree and Principal Components Analysis (PCA) further supported the high reproducibility and low noise achieved in these studies (Fig. 4B, C). These analyses were based on genome-wide gene expression profiles and showed that samples from each brain region formed a cluster distinguishable from those from the other regions, regardless of the experimental animal from which the neurons were derived. This pattern indicated that each homogeneous neuronal population, e.g., the CA1 region neuronal population, had its own distinct transcriptome profile.

**Figure 5:**
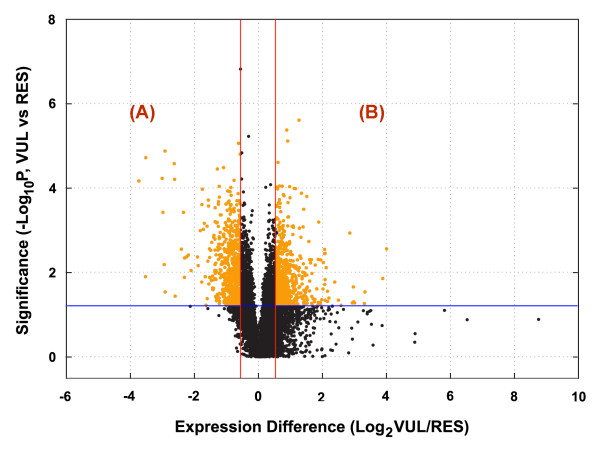
**Identification of differentially expressed genes (orange colored) between the vulnerable (VUL) and resistant (RES) groups by volcano plot**. The Y-axis displays the negative log (base 10) of *P *values from paired *t*-tests, while the X-axis shows the log (base 2) of the -fold differences between the two groups (VUL/RES). The blue line represents the *P *= 0.05 value, and the red lines correspond to fold differences of 1.5 and -1.5, respectively. Genes with paired t-test *P *< 0.05, and fold difference ≥1.5 or ≤-1.5, were identified as differentially expressed genes and are highlighted in orange.

For the planned comparative transcriptome analyses, however, we were interested not in the distinguishing characteristics between one neuronal population and another, as such studies have been performed previously [14-20], but in those characteristics that defined vulnerability *vs*. resistance to OS. Therefore, we classified the neuronal samples into two groups, i.e., neurons vulnerable to OS (CA1 hippocampus and CbG neurons) and those resistant (CA3 hippocampus and cerebral cortex neurons). This approach was expected to focus the analyses of differential gene expression on the common characteristics that might determine sensitivity to OS. Based on the volcano plot analysis shown in Fig 5, there were 994 genes that were differentially expressed in OS-vulnerable as compared with OS-resistant neurons (*t*-test *P *≤ 0.05 and fold difference ≥ 1.5). After adjusting for multiple testing, the Benjamini and Hochberg False Discovery Rate was 0.09. Of the 994 genes, 481 were expressed at significantly higher levels in the OS-resistant neurons (region "A" of Fig. 5), while 513 genes were expressed more highly in OS-vulnerable neurons (region "B" of Fig. 5). Additional file 1 [see Additional file 1] lists the subpopulation of genes in the volcano plot whose expression was ≥ 2.0-fold in vulnerable as compared with resistant neurons, or the reverse. Uncharacterized genes were not included in Additional file 1.

In an effort to confirm that the identified genes were a core set of genes whose expression under basal conditions could differentiate between neurons vulnerable and those resistant to OS, an alternative target-gene identification approach was performed. In this alternative approach, we compared gene expression profiles between CA1 and CA3 and identified differentially expressed genes between them. Likewise, gene expression data generated from CbG and cerebral cortical neurons were also compared and differential gene expression patterns between them were identified. A comparison of convergent genes from the two independent comparisons and the target genes identified using the group-based approach described above (i.e., the 481 genes expressed at higher levels in resistant neurons and the 513 at higher levels in vulnerable neurons) revealed the following: a) 91% of the 481 resistant-neuron-higher genes (a total of 437) were convergent genes that were expressed at higher levels in CA3 and cortex in the two independent comparisons; and b) 94% of the 513 vulnerable-neuron-higher genes (a total of 484) were convergent genes that were expressed at higher levels in CA1 and CbG neurons in the two independent comparisons. These findings suggested that the target genes identified from our group-based approach can be used to differentiate neurons vulnerable to OS from those that are resistant with a high degree of accuracy.

**Figure 6:**
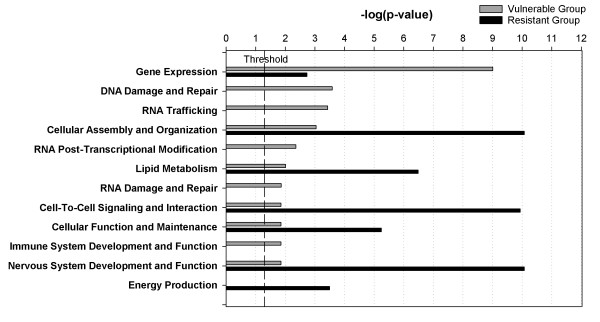
**Comparison of transcriptomic profiles between the VUL and RES neurons based on biological functions associated with the differentially expressed genes identified in each neuronal group**. The threshold line corresponds to the -log value for *P *= 0.05. Values of significance that is higher than threshold indicate greater numbers of genes, from either VUL or RES neurons, associated with the corresponding biological function. For example, although expression of genes related to the biological function of "Gene Expression" reached a value of significance above-threshold for both VUL and RES neurons, there were many more genes expressed in VUL neurons (54 in total) than the RES neurons (2), thus a higher -log *P *value for VUL than RES.

In order to evaluate the potential functional differences between OS-resistant and OS-vulnerable neurons, we performed a comparative analysis on key biological functions associated with the two lists of genes that were expressed more highly either in OS-vulnerable or OS-resistant neurons. The Core Comparison Analysis module in the Ingenuity Pathways Analysis (IPA) package allowed us to gain an overview of key differences between the two types of neurons in terms of their respective gene transcriptomes (Fig. 6). Overall, neurons in the vulnerable group showed higher transcriptional activity for genes related to gene expression, nucleic acid damage repair, RNA trafficking and post-transcriptional modification, and immune response, while those in the resistant group had higher activities in cell signaling, cell function and maintenance, lipid metabolism, and energy production.

We subsequently conducted a series of in-depth bioinformatic analyses of the genes that were differentially expressed in the two populations of neurons. The first of these was the Gene Ontology (GO) characterization, i.e., the determination of biological processes, molecular functions, and cellular components linked to groups of genes with differential expression patterns [28]. In this study, we used a statistical model based on the hypergeometric distribution implemented in MAPPFinder [29]. GO terms enriched with genes which were differentially expressed in vulnerable and resistant neurons are shown in Table 1. A close examination of the results from this analysis and a comparison with those of the IPA analysis showed that there were many common biological functions or processes identified by the two types of analyses. The major functional groups identified by the GO analyses among genes expressed more highly in vulnerable as compared with resistant neurons were: 1) DNA repair and response to DNA damage; 2) Regulation of transcription; and 3) Intramolecular oxidoreductase activity. The GO category of regulation of transcription included a transcription factor that functions as a sensor of hypoxia, hypoxia-inducible factor 1 (*Hif1*), a glucocorticoid receptor, nuclear receptor subfamily 3/group c/member 1 (*Nr3c1*), that controls inflammatory responses in cells, and a transcriptional co-activator of steroid receptors, *Rnpc2*. In addition, one of the transcription factors that transactivates *Nr3c1*, the POU family homeodomain transcription factor *Pou2f1 *[30], was also expressed at higher levels in vulnerable compared with resistant neurons, possibly indicating the importance of inflammatory responses and glucocorticoid receptor signaling in OS-vulnerable neurons. One transcription factor shown in Table 1, chromobox 7 (*Cbx7*), and another listed in Additional file 1 as more highly expressed in OS-vulnerable neurons, MAD homolog 5 (*Smad5*), are involved in maintaining cells in a transcriptionally repressed state and repressing cyclin-dependent cell cycling [31,32]. Elevated expression of genes involved in suppression of cell cycling might be a cell defense against neuronal de-differentiation and cell death [33]. In the last GO category enriched with genes more highly expressed in vulnerable than resistant neurons, there were only two genes, protein disulfide-isomerase A3 precursor (*Pdia3*) and emopamil binding protein (*Ebp*). Both PDIA3 and EBP are isomerases, both may control Ca^2+ ^homeostasis in cells, and both are located in the endoplasmic reticulum (in the lumen or the membrane, respectively) [34,35]. EBP is 3-β-hydroxysteroid-Δ7-Δ8-isomerase that binds drugs that have anti-ischemic properties, such as emopamil and ifenprodil [35]. Based on the differential expression of the genes described above, the general GO category of stress-defense-response genes was identified by GO analysis as being more highly expressed in OS-vulnerable compared with OS-resistant neurons. This GO category is populated by genes in categories of "DNA repair", "response to DNA damage stimulus", and "intramolecular oxidoreductase activity". This is in agreement with the results from the IPA comparative analysis which identified "DNA damage and repair" and "immune system development and function" among the key functions more prevalent in neurons vulnerable to OS.

**Table 1:** Gene Ontology (GO) terms significantly enriched with differentially expressed genes

**GO terms and accession numbers**	Category*	***Z *score**	**Permute *P***	**Associated Genes**
**For genes expressed at higher levels in neurons vulnerable to OS**				

DNA repair (GO:0006281)	BP	4.684	0.001	*Hmgb2, Cspg6, Pold1, Tyms, Xab2*
Response to DNA damage stimulus (GO:0006974)	BP	4.488	0.002	*Hmgb2, Cspg6, Pold1, Tyms, Xab2*
Regulation of transcription (GO:0045449)	BP	2.395	0.011	*Cnot2, Cbx7, Cebpe, Ctcf, E2f5, Gmeb2, Hif1a, Nfyc, Nr3c1, Pnrc1, Pou2f1, Rere, Rnpc2, Usf2, Zfp57*
Intramolecular oxidoreductase activity (GO:0016860)	MF	3.165	0.040	*Pdia3, Ebp*

**For genes expressed at higher levels in neurons resistant to OS**				

Calcium- and calmodulin-dependent protein kinase activity (GO:0004685)	MF	5.475	0	*Camk2a, Camkk1, Camk1b*
Energy derivation by oxidation of organic compounds (GO:0015980)	BP	2.839	0.004	*Idh3a, Gyg1, Eno1, Pfkp, Ldha, Me1, Idh1*
Neurotransmitter transport (GO:0006836)	BP	3.313	0.008	*Cplx2, Rims2, Slc6a7, Slc6a9, Stx1a*
Main pathways of carbohydrate metabolism (GO:0006092)	BP	2.739	0.010	*Eno1, Pfkp, Ldha, Idh3a, Me1, Idhc*
Synaptic vesicle (GO:0008021)	CC	2.96	0.012	*Sept5, Syn2, Syp, Syt5*
Regulation of neurotransmitter secretion (GO:0046928)	BP	3.902	0.022	*Kcnmb4, Syn2*
Regulation of action potential (GO:0001508)	BP	3.902	0.022	*Kcnmb4, Pllp*
Regulation of signal transduction (GO:0009966)	BP	2.858	0.025	*Igfbp3, Rgs14, Rgs3, Socs2*

* BP – Biological Process; CC – Cellular Component; MF – Molecular Function

The GO categories that contained genes with higher expression in OS-resistant *vs*. OS-sensitive neurons were those of energy generation and carbohydrate metabolism, as well as action potential, neurotransmission, calcium signaling, and regulation of signal transduction. Related GO terms such as "calcium- and calmodulin-dependent protein kinase activity", "regulation of signal transduction", "neurotransmitter transport", "synaptic vesicle", "regulation of neurotransmitter secretion", and "regulation of action potential" were enriched with genes expressed at higher levels in OS-resistant than OS-vulnerable neurons. The GO terms "energy derivation by oxidation of organic compounds" and "main pathways of carbohydrate metabolism" were also enriched with genes that were expressed at higher levels in resistant as compared with vulnerable neurons. These results matched some of the key categories identified through the use of the IPA analysis, in particular, the two major categories of genes expressed more highly in OS-resistant than OS-vulnerable neurons, "cell-to cell signaling and interaction" and "energy production".

In the present studies, OS-resistant neurons had significantly lower transcription levels of genes that fell in the GO categories of "defense response" (GO:0006952, *Z *= -2.14, *P *= 0.027) and "immune response" (GO:0006955, *Z *= -2.037, *P *= 0.043) compared with OS-vulnerable neurons. These results are not shown in Table 1 as only GO terms with positive *Z *scores are included in that table. The negative *Z *scores associated with the GO terms "defense response" and "immune response" might indicate that either the genes in these two categories were down-regulated in resistant neurons or they were more highly expressed in vulnerable neurons.

To explore further the biological significance of differences in gene expression between OS-vulnerable and OS-resistant neurons, the patterns of differential gene expression were analyzed to determine the role of such genes in defined biological pathways. Using a statistical model similar to the GO analysis, biological pathway analysis assembles genes (or their corresponding proteins) into categories of functions that fit certain biological tasks or processes. Our analysis covered nearly all the currently known biological pathways deposited in various databases, including the Kyoto Encyclopedia of Genes and Genomes (KEGG) [36], BioCarta [37] and GenMAPP [38]. Although this approach is different from that of the IPA and GO analyses, the results of pathway analysis (Table 2) showed substantial agreement with those of IPA and GO analyses.

**Table 2:** Biological pathways significantly enriched with differentially expressed genes

**Biological pathways**	***Z *score**	**Permute *P***	**Associated Genes**
For genes expressed at higher levels in neurons vulnerable to OS			

mRNA processing reactome	5.708	0	*Cugbp1, Dhx16, Hnrpa1, Hnrpa3, Hnrph1, Hnrpu, Mettl3, Nsep1, Rnpc2, Sfrs3, Sf3b1*
Pyrimidine metabolism	3.921	0.008	*Nt5e, Pold1, Tyms*
Alanine and aspartate metabolism	3.265	0.021	*Gpt1, Got2*

**For genes expressed at higher levels in neurons resistant to OS**			

MAPK cascade	6.016	0	*Araf, Hras, Map2k1, Mapk1, Mapk10, Mbp, Jun*
Citrate (or TCA) cycle	3.897	0.006	*Acly, Idh1, Idh3a*
Regulation of actin cytoskeleton	2.524	0.014	*Enah, Fgf13, Fgfr2, Chrm3, Gna12, Hras, Map2k1, Mapk1, Pak3, Pip5k1c, Slc9a1*
Prostaglandin synthesis regulation	3.315	0.015	*Anxa4, Anxa5, Anxa6, Ptgs2, Hpgd*
Wnt signaling	2.882	0.020	*Ccnd2, Jun, Mapk10, Prkce, Wnt2b*
Insulin signaling	2.136	0.035	*Cap1, Gyg1, Hras, Igf1r, Jun, Map2k1, Mapk1, Mapk10, Pten, Sgk*
G protein signaling	2.154	0.041	*Akap13, Akap6, Gna12, Gng8, Hras1, Prkce, Slc9a1*

The pyrimidine metabolism pathway, enriched with genes expressed more highly in OS-vulnerable compared with OS-resistant neurons, and the citrate (or TCA) cycle biological pathway, enriched with genes more highly expressed in OS-resistant than OS-vulnerable neurons corresponded, to some extent, to the theme of DNA damage/repair and energy derivation/carbohydrate metabolism shown in Fig 6 and table 1. The biological pathway analyses, however, revealed some new pathways, such as the alanine/aspartate metabolism pathway that was enriched with genes more highly expressed in OS-vulnerable *vs*. OS-resistant neurons, and the signal transduction, prostaglandin synthesis, and actin cytoskeleton regulation pathways that were enriched with genes expressed more highly in OS-resistant *vs*. OS-vulnerable neurons (Table 2). The signal transduction pathways included the MAPK cascade, Wnt signaling, insulin signaling, and G-protein signaling pathways. Some of the genes in the biological pathways listed in table 2 may also be involved in cell signaling and may have a neuroprotective function, for example, the annexin IV (*Anxa4*), annexin V (*Anxa5*), and annexin VI (*Anxa6*) genes [39-41].

Pathway analysis identified four genes belonging to the protein ubiquitination pathway as expressed more highly in OS-vulnerable than OS-resistant neurons. This pathway did not reach statistical significance due to the large number of genes involved. Nevertheless, the four genes whose expression was high in OS-sensitive neurons, *Usp1 *(ubiquitin specific peptidase 1, VUL/RES = 10.08, *P *= 0.029; VUL: vulnerable group [CbG + CA1], RES: resistant group [Cortex + CA3]), *Usp3 *(ubiquitin specific peptidase 3, VUL/RES = 4.63, *P *= 0.040), *Usp48 *(ubiquitin specific peptidase 48, VUL/RES = 1.63, *P *= 0.001) and *Ube2e2 *(ubiquitin-conjugating enzyme E2E 2, VUL/RES = 1.54, *P *= 0.049), may be indicators of high stress in vulnerable neurons.

The biological function of energy generation was populated by genes expressed more highly in OS-resistant than OS-vulnerable neurons. We performed gene networking analyses on genes related to cell energy generation using information from currently available literature data [see Additional file 2]. This *in silico *network construct identified several of the energy-generating genes in the network that were expressed more highly in neurons from regions resistant to OS. These genes included *Eno1*, *Idh1*, *Idh3a*, *Ldha*, *Me1*, and *Pfkp*. Genes that interact with *Eno1*, *Idh1*, *Idh3a*, *Ldha*, *Me1*, and *Pfkp *were identified by gene networking analyses and included *Cap1, Egf, Grem1 *and *Tgfb1*. The function of these five genes is related to cell development, including differentiation and proliferation, and cell-to-cell signaling and interaction. The gene network analysis suggested a link between energy production and the high energy demand for cell signaling in OS-resistant neurons. Direct experimental evidence on differential levels of energy reserves, i.e., ATP levels, in OS-vulnerable and OS-resistant neurons is presented below.

### Comparison of gene expression *vs*. protein levels of SOD1, SOD2 and GPX1

The expression of genes for cellular oxidoreductases known to protect neurons and other cells from OS, i.e., those for *Sod1*, *Sod2*, or *Gpx1*, was examined for possible differential patterns of transcriptional regulation of these enzymes. The microarray data on *Sod1*, *Gpx1*, and *Sod2 *indicated no significant differences in expression between CbG and cerebral cortical neurons. The lack of differential expression of the genes for *Sod1 *and *Gpx1 *was anticipated based on the lack of any differences in SOD1 and GPX1 protein levels between CbG and cerebral cortical neurons in culture in the absence of added OS stimuli. But, whereas the protein levels of SOD2 were 2.5 times higher in CbG compared with cerebral cortical neurons in cultures without the added stimulus of paraquat-induced OS, the *Sod2 *gene levels were only marginally higher in OS-vulnerable compared with OS-resistant neurons captured from fresh brain sections by LCM (VUL/RES = 1.16, *P *= 0.063). Thus, the higher protein expression levels of SOD2 in CbG neurons in culture might have been the result of exposure of primary neurons to the increased O_2 _tension in culture.

### Confirmation of GeneChip data with real-time quantitative PCR

**Figure 7:**
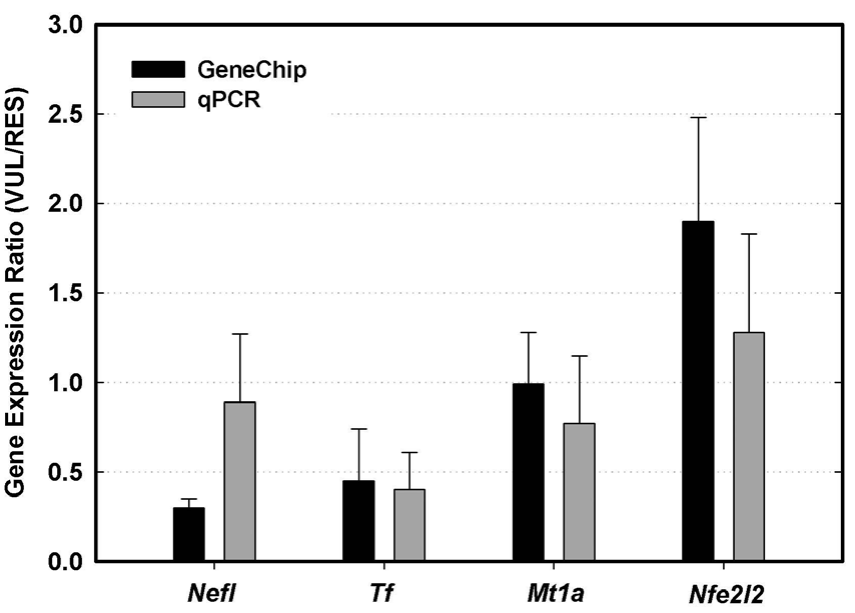
**Validation of microarray data by real-time qPCR**. Shown are the average ratios (± SEM, n = 3) of expression of the genes *Mt1a*, *Nefl*, *Nfe2l2*, and *Tf*, in VUL *vs*. RES neurons as measured by GeneChip and qPCR. The qPCR measurements are not significantly different from the GeneChip data (*P *values range: 0.199 – 0.896).

The results of the microarray analyses were validated by real-time quantitative PCR (qPCR) measurements of the expression of select genes. These genes were: *Nefl*, neurofilament, light polypeptide; *Tf*, transferrin; *Mt1a*, metallothionein 1a; and *Nfe2l2 *(or *Nrf2*), nuclear factor, erythroid derived 2, like 2. The selection of these genes was based on two criteria: 1) their expression profiles were representative of most genes on the array in terms of the range of transcript abundance levels and expression ratios between VUL and RES neurons; and 2) the genes were considered important in terms of differential vulnerability of cells to OS [20]. For all selected genes, the qPCR data confirmed the relative expression ratios in VUL *vs*. RES neurons (Fig. 7). Among the four genes, three (*Mt1a*, *Nfe2l2*, and *Tf*) showed similar ratios of expression as determined by microarray and qPCR. For *Nefl*, the array analysis showed a ratio of 0.30 (VUL/RES) whereas the ratio estimated by qPCR indicated only slightly lower expression in VUL as compared with RES neurons (VUL/RES = 0.89). This difference might have been due to the fact that the targeted gene regions for detection by the two approaches were different. The GeneChip probe set targeted the last exon (exon 4) of *Nefl*, while the qPCR analysis, because of constraints in primer design, targeted exons 1–2. Despite this difference, the patterns of expression of the four genes estimated by qPCR were consistent with those estimated by microarray analyses.

### Comparative levels of ATP generation in CbG and cortical neurons in primary cultures

**Figure 8:**
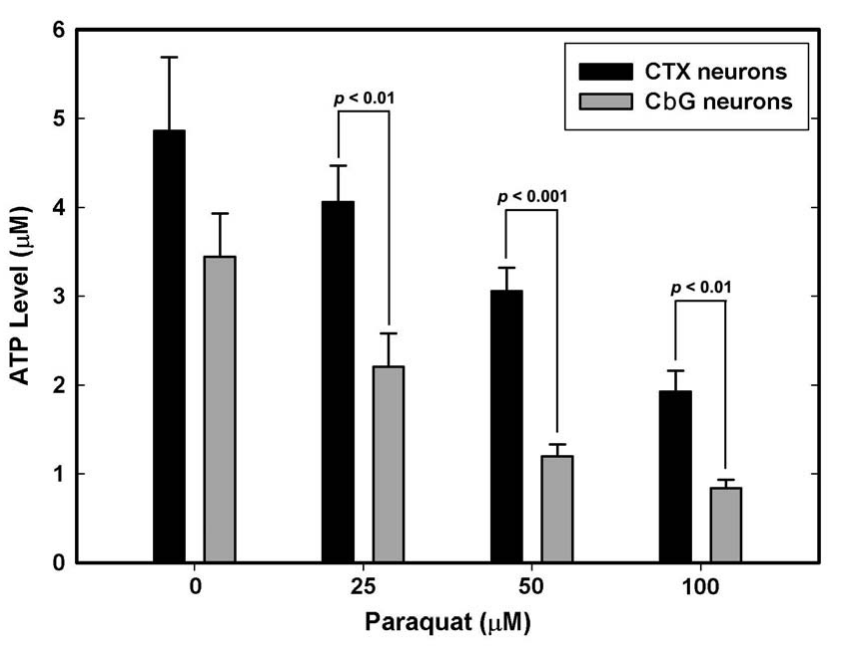
**Differential levels of ATP and the effect of paraquat on these levels in CTX and CbG neurons**. Data represent the mean (± SEM) of ATP levels from 8 experiments. The *P *values for the indicated comparisons (*t*-test) are shown.

The results of genomic analyses indicated that neurons vulnerable to OS had a lower expression of genes involved in energy generation. To determine if this would be true in neurons maintained in culture, we measured the levels of ATP in primary CbG and cerebral cortical neurons, both before and after OS induction. The ATP levels of CbG neurons under basal conditions were approximately 25% lower than those of cerebral cortical neurons, but this difference was not statistically significant. However, when exposed to OS, ATP levels in both cell types dropped, but they did so more precipitously and significantly in CbG than cortical neurons (Fig. 8). Two-way ANOVA on the combined data of all paraquat concentrations showed that, overall, CbG neurons had significantly lower ATP levels than cortical neurons (*df *= 1, *F *= 16.677, *P *< 0.001). In addition, the ANOVA results demonstrated that paraquat had a significant effect on ATP levels (*df *= 3, *F *= 9.513, *P *< 0.001). To explore further the relationship between OS and ATP levels in these two populations of neurons, we measured changes in the levels of ATP in CbG and cortical neurons under two different levels of O_2 _tension (5% and 20% O_2_). While the ATP levels in cerebral cortical neurons were not significantly altered by growing them for 7 and 14 DIV under 20% O_2 _tension, the levels dropped to significantly lower values (*P *< 0.05) in neurons maintained for 21 DIV under high O_2 _tension when compared with those maintained under 5% O_2 _(data not shown). CbG neurons were more sensitive to the different levels of oxygen and showed significant decreases in ATP levels at both 14 and 21 DIV for cells exposed to 20% O_2 _tension compared with those maintained at 5% O_2 _(*P *< 0.05 and *P *< 0.01 for 14 and 21 DIV neurons, respectively; data not shown). Two-way ANOVA on combined data of cerebral cortical and CbG neurons demonstrated, once again, that cerebral cortical neurons had significantly more ATP than CbG neurons (*df *= 1, *F *= 12.089, *P *= 0.001).

## Discussion

Differential sensitivity of neurons to OS was shown in the present study to be a property of four different neuronal populations, hippocampal pyramidal neurons from two adjacent regions, CA1 and CA3, and neurons from two distant regions of the brain, CbG and cerebral cortex neurons. Selective neuronal vulnerability has been observed in many previous studies in association with neurodegenerative diseases, such as Alzheimer's disease that affects primarily neurons in the cerebral cortex, hippocampus and amygdala [42,43], Parkinson's disease that is associated with the death of dopaminergic neurons of the substantia nigra [44], and amyotrophic lateral sclerosis that is related to degeneration of cortical, brain stem and, especially, spinal motor neurons [45]. In addition, within a single brain region, such as the human or rodent hippocampus, selective vulnerability of CA1 *vs*. CA3 neurons has been observed following episodes of global cerebral ischemia [11,46], early phases of Alzheimer's disease [47], chronic epileptic seizures [48], and OS [5,20]. OS is a common denominator among these various adverse conditions. For this reason, in the present study, as well as in our previous studies, we focused on the selective vulnerability of neurons to OS.

There are only a few studies that have compared the sensitivity of CbG to cerebral cortical neurons. Oxygen and glucose deprivation followed by re-oxygenation revealed a much greater susceptibility of CbG than cerebral cortical primary neurons to these stressful conditions, and the same is true for the toxicity induced by methyl-mercury treatment of primary neurons [49,50]. Oxygen and glucose deprivation also caused greater suppression of ATP levels in CbG than cortical cells. The effects of these manipulations were attributed to a higher sensitivity of CbG neurons to OS as compared with cerebral cortical cells. For example, methyl mercury exposure leads to the generation of higher levels of intracellular ROS in CbG than in cortical neurons [49] and ischemia/re-oxygenation is known to produce OS. Based on the observations made in the present study with primary neuronal and organotypic slice cultures, as well as those in the studies cited above, there appears to be marked differential susceptibility of CbG and cerebral cortical neurons to OS. In the present study, we found that CbG neurons exhibited significantly lower viability in primary cultures than cerebral cortical neurons, even prior to OS treatment. The difference in neuronal viability between the two populations of neurons might have been related to an intrinsically high stress response of CbG neurons. Nevertheless, the lower survival rate of CbG neurons even in the absence of OS was not viewed as an invalidation of the findings that CbG neurons were more vulnerable to OS than cortical neurons. The differential vulnerability of CbG neurons to OS induced by paraquat when compared with that of cortical neurons was demonstrated by the fact that the rate of cell loss was greater in CbG than cerebral cortical neurons at every paraquat concentration to which the neurons were exposed (Fig. 1A).

The differences in responses to OS between CbG and cerebral cortical neurons were observed under strong conditions of OS, such as exposure to paraquat (present study), treatment with methyl mercury [49], or exposure to oxygen-glucose deprivation and re-oxygenation [50], as well as under milder stimuli of OS, i.e., growth of neurons under 20% O_2 _tension. All previous studies comparing relative CbG and cortical neuron susceptibility to OS were performed with neuronal cultures maintained under ambient atmospheric, i.e., 20% O_2 _tension [49,50]. But, as we showed in the present study, exposure of CbG neurons, in particular, to this level of O_2 _tension produces cellular OS that leads to reduced survival of neurons in culture. Future studies examining differences in responses to OS and the molecular processes that determine differential sensitivities to OS, should be performed with neurons that were grown under low O_2 _tension.

Since OS plays an important role in the aging process, the study of the fate of CbG neurons during the aging process might be viewed as a model of possible selective vulnerability of these neurons to OS. There is evidence that the cerebellum granule cell layer undergoes both a loss of CbG neurons as well as degenerative changes in their structure as a result of aging. Stereological studies of human cerebellum have shown that the number of CbG neurons is significantly decreased (around 40%) in aged individuals [51,52]. Moreover, there is a notable diminution of cell volume, axons and synapses in the remaining neurons [52]. A similar trend has been observed in other mammalian brains as well [53] and such cell losses and structural changes may be related to the poor motor coordination, impaired motor learning, and loss of muscular tone that occur with advancing age.

Our initial studies of protein expression levels in CbG and cerebral cortical neurons in culture failed to identify a causal relationship between enzyme levels for cellular oxidoreductases and CbG neuron susceptibility to OS. Specifically, CbG neurons expressed higher levels of SOD2 and GPX1 than cerebral cortical neurons did in response to increases in OS, yet CbG cells were not better protected from OS. Whatever molecular differences exist between these two types of neurons to account for their differential vulnerability to OS, the differences could not be overcome by the greater increases in OS-induced SOD2 and GPX1 levels in CbG neurons. These results indicate that it is the expression of genes and gene products other than general oxidoreductases that impart either enhanced susceptibility of CbG cells or increased resistance of cortical neurons to OS. If this interpretation is valid, then other populations of OS-vulnerable and OS-resistant neurons, such as the CA1 and CA3 neurons of the hippocampus, might exhibit some patterns of gene expression that are similar to those of CbG and cortical neurons.

The transcriptomic analyses performed on CbG, CA1, CA3 and cerebral cortical neurons that were captured from brain sections under basal conditions, revealed that each neuronal population expressed distinguishable clusters of genes. Nevertheless, by combining the gene expression patterns of neurons that were shown to be vulnerable to OS and comparing these patterns to those of neurons that were resistant to OS, we were able to identify key biological functions, GO categories and pathways that characterized vulnerable *vs*. resistant neurons. Importantly, these differential patterns of gene expression represented endogenous neuronal differences, not differences brought about by exposure of these populations of neurons to any form of OS. The key differences could be summarized as follows. Neurons that are OS-vulnerable expressed higher levels than did neurons resistant to OS of genes involved in cell stress defense, suppression of transcriptional activity, repair of DNA damage, control of cell cycling, steroid receptor signaling, and maintenance of intra-endoplasmic reticulum reducing conditions and Ca^2+ ^levels. On the other hand, OS-resistant neurons expressed more highly than OS-vulnerable neurons genes related to neurotransmission, action potential conduction, signal transduction, and energy generation.

In our previous study of differential responses of CA1 and CA3 neurons to OS, we observed that during the course of exposure of neurons to OS, CA1 neurons had consistently higher expression than CA3 neurons of genes in the categories of stress/inflammatory response, transition metal transport, ferroxidase, and presynaptic signaling activity [21]. On the other hand, CA3 neurons had higher expression than CA1 neurons of genes in the categories of GABA signaling, postsynaptic signaling, and calcium and potassium channel activity [21]. The main goal of the present study was to probe for intrinsic, rather than OS-induced, biochemical and transcriptional differences between vulnerable (CA1 + CbG) and resistant (CA3 + cortical) neurons from four brain regions. Unlike the previous study of transcriptomic analysis of CA1 and CA3 neurons, there was no OS applied to the neurons sampled for the transcriptomic analyses, the analyses were extended to neurons from other regions with differential sensitivities to OS, and the neurons were captured from brain sections as proximal to the *in vivo *condition as possible. Nevertheless, despite the differences between the conduct of the two studies, some of the findings from the current study were in accordance with the results from the previous study. In the GO analysis, sensitive neurons had higher transcriptional activity than resistant neurons of stress response genes; resistant neurons had relatively higher expression of genes related to signal transduction and neurotransmission than vulnerable neurons. This consistency indicated that these key transcriptional differences are potentially good markers in differentiating neurons that are vulnerable to OS from those that are resistant. At the level of differences in expression of single genes, of the 31 genes that were more highly expressed in CA3 than CA1 during induction of OS, 18 were genes that were also expressed at significantly higher levels in the OS-resistant *vs*. OS-vulnerable neurons in the present study (the known genes are listed in Table 3). Included among these genes were (also listed in Additional file 1): *Nefl*, neurofilament light polypeptide; *Gabra5*, γ-aminobutyric acid receptor 5 α; *Rab15*, member of *Ras *oncogene family; *Opcml*, opioid binding protein/cell adhesion molecule-like; *Pllp*, plasma membrane proteolipid; *Cadps*, Ca^2+^-dependent secretion activator; *Ncald*, neurocalcin δ; and *Cacna2d3*, calcium channel (voltage gated) α2/δ3 subunit.

**Table 3:** Currently known genes that were more highly expressed in resistant than vulnerable neurons in both the present and previous study [21]

**Affymetrix_ID**	**Gene Symbol**	**Description**	**Fold Difference (RES/VUL)^a^**	***t*-Test *P *Value^b^**
1372953_at	*Ncald*	neurocalcin delta	7.90	3.79E-04

1370058_at	*Nefl*	neurofilament, light polypeptide	3.28	9.53E-04

1390358_at	*Cacna2d3*	calcium channel, voltage-dependent, alpha 2/delta 3 subunit	2.91	0.019

1368810_a_at	*Mbp*	myelin basic protein	2.33	0.011

1373646_at	*Rab15*	RAB15, member RAS onocogene family	2.17	2.22E-03

1371057_at	*Gabra5*	gamma-aminobutyric acid (GABA-A) receptor, subunit alpha 5	2.14	0.019

1368114_at	*Fgf13*	fibroblast growth factor 13	2.13	1.26E-03

1368523_at	*Cadps*	Ca2+-dependent secretion activator	1.86	3.31E-03

1386943_at	*Pllp*	plasma membrane proteolipid	1.59	0.025

1368861_a_at	*Mag*	myelin-associated glycoprotein	1.56	0.024

1387961_at	*Opcml*	opioid binding protein/cell adhesion molecule-like	1.50	4.37E-04

^a,b ^– Calculations based on data from this study. Since the previous study [21] was a time-course study, no corresponding data were available.

Some of the pronounced findings (higher stress and lower energy production in vulnerable neurons) generated from the present study of functional genomics, were directly supported by traditional biochemical data. In our previous reports, we demonstrated the existence of higher intrinsic stress and inflammatory response in the vulnerable CA1 neurons [20,21]. The *in vitro *measurement of basal superoxide generation in the hippocampal CA1 and CA3 regions showed that the vulnerable CA1 neurons generated more superoxide than the resistant CA3 neurons. In the present report, the *in vitro *measurements of ATP levels in CbG and cerebral cortical neurons in culture confirmed the differential levels of energy generation in these two cell populations.

Furthermore, these studies confirmed previous observations that the depletion of ATP stores in CbG neurons is much greater than that in cerebral cortical neurons upon exposure to OS [50]. To survive stressful conditions, neurons would need readily available sources of energy, mostly in the form of ATP. ATP is needed for repair or replenishment of damaged cellular components, such as DNA and proteins, and for re-establishing ionic gradients. Studies on the selectively vulnerable dopamine neurons of the substantia nigra indicate that lower ATP production and higher OS may be consequences of mitochondrial dysfunction [54]. Mitochondrial complex I activity in neurons of the substantia nigra obtained from patients suffering from the sporadic form of Parkinson's disease is decreased [55]. Mitochondrial complex I defects may contribute to decreases in ATP synthesis and to excesses in the production of ROS in nigra neurons. Further support for the idea that OS-vulnerable neurons may be subjected to increases in ROS formation throughout their lifespan comes from observations that mitochondria isolated from CA1 neurons of the hippocampus release more ROS than those from neurons of the CA3 region [56].

It is important to point out that four major gene expression patterns characteristic of neurons vulnerable to OS, i.e., high stress and immune responses, decreased energy generation, increased DNA repair, and reduced signal transduction, are similar to gene expression profiles that have been observed in cells from aging brain [57-62]. The increases in expression of some of these genes, for example, those related to DNA repair, have been linked to increases in OS in neurons, to aging-associated neuronal gene expression, and to DNA damage in genomic DNA of cells in the brains of aged individuals as a result of nucleotide base oxidation [60,61]. In addition to the increases in the gene categories described above, there are decreases in aging brain of genes related to neurotransmission and signal transduction [61]. The similarities in transcriptome patterns between neurons that are vulnerable to OS and those that have gone through the aging process in brain may indicate that aging-related events enrich the gene expression patterns that are seen in neurons that are vulnerable to OS. Such enrichment of gene expression may have important adaptive functions for the aging brain, such as defense against age-associated increases in OS-induced damage.

## Conclusion

Oxidative stress plays an important role in brain aging and neurodegenerative diseases but not all brain neurons are equally sensitive to OS. Those that are sensitive to OS are the first ones to suffer functional decline during aging or cell death in neurodegenerative diseases. To protect these vulnerable neurons from age- or disease-associated injury, the first step to be accomplished is to understand the molecular mechanisms that underlie the high sensitivity of certain neurons to OS. However, the molecular determinants of differential neuronal sensitivity to OS remain unknown. In this study, the integration of high-precision target neuron collection by LCM, high-throughput functional genomics, and more targeted biochemical analyses were employed to improve our understanding of SNV to OS. The study of more than one population of sensitive and resistant neurons was deliberately introduced in order to increase the confidence in the results obtained. The data provided evidence that high stress levels and low energy reserves were important predisposing factors of SNV to OS. Thus, potentially, vulnerable neurons even under normal conditions are under elevated levels of stress. The increases in OS resulting from the aging process or from the onset of a neurodegenerative disease may shift the balance between stress and cell defense mechanisms and cause these neurons to die more readily than resistant neurons. The lack of energy reserves would directly compromise the defensive responses of neurons vulnerable to OS and increase the probabilities of neuronal injury under conditions of OS. Therefore, potentially useful approaches to protecting vulnerable neurons during aging or in disease states, such as Alzheimer's disease, would be those that are focused on decreasing endogenous levels of OS, increasing energy metabolism, or enhancing some of the cell defense processes that we found to be up-regulated in vulnerable neurons.

## Methods

### Primary neuronal cultures and OS induction

Dissociated cerebral cortex and CbG neuron cultures were established as described before [63-65]. All animal procedures were performed in accordance with guidelines established by the University of Kansas IACUC. For preparations of cortical neuron cultures, the cortical lobes from 18-day old Sprague Dawley fetuses were dissected and cells dissociated by gentle trituration with trypsin and harvested by centrifugation. Cortical cells were re-suspended in fresh DMEM/F-12 with 10% fetal calf serum (FCS), plated at densities ranging from 0.3 to 3 × 10^6 ^cells/35 mm dish, coated with poly-D-lysine, and after 24 h, the FCS-containing medium was replaced by a defined medium with DMEM/F12 containing N_2 _supplements, KHCO_3 _(15 mM), and 20% glial conditioned medium. CbG neurons were prepared from 7–8 day old Sprague Dawley pups. The dissociated cells were re-suspended in Krebs-Ringer-buffered medium. They were then treated with trypsin (50 μg/ml) and subsequently trypsin inhibitor and DNase (80 μg/ml), plated at densities ranging from 0.3 to 3 × 10^6 ^cells/35 mm dish (coated as above) and maintained in basal Eagle's medium supplemented with 10% FCS, 25 mM KCl, 2 mM glutamine, and 100 μg/ml gentamicin. Growth of glial cells was inhibited by the addition of 10 μM cytosine arabinoside. These cultures were maintained at 37°C in ambient air mixed with 5% CO_2_.

OS was induced by exposing primary cortex and CbG neurons to increasing concentrations of paraquat (1,1^'^-dimethyl-4,4^'^-bipyridynium dichloride; 0–100 μM). Neurons plated at 6 × 10^5 ^cells/35 mm dish were exposed to freshly prepared paraquat added to the culture medium. After 24 h, neuronal viability was determined by the calcein AM and propidium iodide (PI) labeling method [64]. The medium was removed, replaced with 1 ml PBS containing 150 nM calcein AM and 20 μM PI for 30 min, after which the cells were rinsed with PBS and visualized in a Nikon fluorescence microscope. Viability was determined by counting the number of green (alive) and red (dead) cells. Neurons were classified as vulnerable if after treatment their survival rate dropped below 40%.

### Neuronal cultures under different O_2 _tension

To test the effects of O_2 _tension on CbG and cortical neurons, primary neuron cultures established in the same way as described above were maintained in either 5% or 20% O_2_, in 90% or 75% N_2_, respectively, and in a constant 5% CO_2 _atmosphere. Cell viability was measured by the 3-(4,5-dimethylthiazol-2-yl)-2,5-diphenyltetrazolium bromide (MTT) reduction assay [66]. The assay was performed by incubating neuronal cultures with MTT (0.5 mg/ml) for 4 h, solubilizing the formazan crystals formed by the addition of an equal volume of 0.04 N HCl in isopropanol, sonicating the samples (2 min), and measuring the absorbance at 570 nm. Background absorbance was determined in cells pre-treated with iodoacetamide (10 mM), an inhibitor of succinate dehydrogenase.

### Biochemical analyses

For immunoblot studies, proteins were extracted from primary neurons 24 h after exposure to either paraquat or buffer. Cells were lysed in 3 mM Tris-Cl, pH 8.0, containing 10 μl/ml of a protease inhibitor cocktail (Calbiochem, San Diego, CA, USA) and the extracted proteins were separated by SDS-PAGE, transferred to PVDF membranes, and probed with antibodies to cytochrome P450 reductase (1:200) (Nventa, San Diego, CA, USA), Cu/Zn-superoxide dismutase (SOD1) (1:1000) (Chemicon, Temecula, CA, USA), Mn-SOD (SOD2, 1:1000) (Stressgen, Victoria, Canada), and glutathione peroxidase 1 (GPX1, 1:500) (Cortex Biochem, San Leandro, CA, USA). Equal amounts of protein sample were loaded into each lane of SDS-PAGE gels. Incubation with alkaline phosphatase-conjugated secondary antibody (1:1000) and color development were as described [67]. Immunoblots were scanned and densitometric analyses performed using Photoshop 5.0.

The levels of cellular ATP were measured using the ATP Determination Kit (Molecular Probes, Carlsbad, CA, USA) according to the manufacturer's instructions. The assay is based on the generation of light by the ATP-dependent reaction between luciferin and luciferase. The reaction mixture in a final volume of 100 μl contained: 25 mM Tricine buffer, pH 7.8, 0.1 mM EDTA, 0.5 mM luciferin, and 1.25 μg/ml luciferase. The reaction was initiated by the addition of 10 μl of neuronal lysate prepared from control and paraquat-treated cells, and the resultant bioluminescence (emission maximum 560 nm) measured in a luminometer. The concentration of cellular ATP was calculated by a standard curve generated using known concentrations of exogenous ATP.

### Organotypic brain slice cultures and induction of OS

Two types of brain slice preparations were employed in the studies: acute slice preparations from adult rat brains and organotypic slice cultures from newborn rats. The acute slice preparations were obtained from 3 mo-old male rats, whereas the organotypic slice cultures were established from 10-day old male rats [20,21,68]. For the organotypic cultures, slices (300 μm thick) of hippocampus, cerebellum, and cerebral frontal cortex were prepared and maintained in culture for one week in 50% MEM, 25% Hanks' balanced salt solution, and 25% heat-inactivated horse serum, plus 4.5 mg/ml glucose, 1 mM glutamine, and 20 ml/L penicillin-streptomycin. The culture medium was removed 24 h prior to treatment with paraquat and was replaced with serum-free medium. Slices were either treated with 100 μM paraquat for 1 h (OS) or buffer (controls). Neuronal death was determined at 24 h after initiation of treatment by staining with PI. PI was added to the treatment medium at a final concentration of 5 μg/ml. PI uptake was used to monitor cell death since it is excluded from live cells. Fluorescence from the PI staining was excited at 515–560 nm using a Zeiss microscope fitted with rhodamine filter. Fluorescence as well as visible-light (to visualize the whole area) images were captured, and the percentage of dead neurons was calculated. For the acute slice preparations, the slices from the same brain regions as those used for organotypic cultures were incubated in serum-free medium (described above) for 2 h to allow for recovery from the dissection. Subsequent paraquat treatment and estimation of cell death by measuring PI uptake, were performed the same as for the above organotypic cultures.

### Cryosectioning and laser capture microdissection (LCM)

An optimized protocol [69] from Arcturus (Mountain View, CA, USA) was followed for LCM. Six-months-old Fisher 344/BN F1 rats that were not subjected to any prior treatment were sacrificed, the brain dissected and immediately frozen in liquid N_2_, and cryosections (8 μm thick) obtained. In order to preserve RNA quality, frozen sections were quickly stained with Arcturus HistoGene LCM Frozen Section Staining Kit. The Arcturus PixCell^® ^IIe Laser Capture Microdissection System was used for capturing the cells. The four populations of target neurons were collected from the hippocampal CA1 and CA3 regions, the CbG cell layer, and the frontal cortex (layers IV-VI).

### RNA extraction and GeneChip hybridization

Total RNA was extracted from the microdissected neurons with the Arcturus PicoPure RNA isolation kit. Three replicate samples representing each of the four neuronal populations from each animal were obtained. The quality of the RNA samples was checked with Agilent Bioanalyzer 2100 with RNA 6000 Pico Chips (Agilent Technologies, Santa Clara, CA, USA). For GeneChip target preparation with signal amplification, the GeneChip Two-Cycle cDNA Synthesis Kit from Affymetrix (Santa Clara, CA, USA) was used according to the manufacturer's instructions. The Affymetrix Rat Expression 230A (RAE230A) oligonucleotide arrays containing 14,280 Unigene clusters were used for the hybridization at 45°C for 16 h. Subsequent washing and staining steps were performed on an Affymetrix GeneChip System running GeneChip Operating Software (GCOS, ver 1.1.1). In order to minimize experimental variability, all steps throughout the entire process, from cryosectioning to array data collection, were performed by the same investigator. The microarray data generated from all chips met the quality control criteria set by Affymetrix, including low background and noise, positive detection of QC probesets such as bioB, percentage of genes called present in normal range (generally between 40–60%), similar scaling factors across all chips, and 3'/5' ratio. All microarray data were deposited in NCBI's Gene Expression Omnibus [70], with series accession number GSE7139.

### GeneChip data analysis

The microarray data generated from GCOS, scaled to the same signal intensity of 500, were imported to GeneSpring (version 7.2) (Agilent Technologies, Santa Clara, CA, USA). In GeneSpring, the data were normalized on a Per Chip (to 50^th ^percentile) and Per Gene (to median) basis. Gene Tree and PCA were subsequently conducted to examine the reproducibility of samples from each brain region. Both analyses were based on all probesets on the array and Pearson correlation was used to measure similarity.

For identification of differentially expressed genes between vulnerable and resistant neurons, genes showing expression (i.e., called "present" by GCOS) in at least two thirds of all samples were selected as candidate genes, while the rest were filtered out from further analysis. For subsequent data analysis, samples from hippocampal CA1 and CbG neurons were classified as the vulnerable group (VUL), and those from CA3 and cortex neurons as the resistant group (RES). In order for a gene to be identified as differing significantly in expression between the two groups of neurons, two criteria had to be met: 1) average VUL/RES ratio of gene expression had to be either ≥1.5 or ≤0.667, i.e., genes had to have at least a 1.5-fold higher expression in the VUL over RES, or a 1.5-fold higher expression in the RES over VUL group; and 2) differential gene expression had to reach statistical significance of *P *≤ 0.05 in paired *t*-test statistical comparisons. Genes fulfilling both criteria were plotted in a volcano plot. Multiple testing corrections were based on the Benjamini and Hochberg False Discovery Rate approach [71].

Genes showing significantly higher expression in VUL or RES neuronal populations were subjected to further comparative analyses of their overall biological functions. GO, Biological Pathway and gene networking analyses were used to uncover their functional profiles. Comparative analyses of the biological functions of differentially expressed genes in VUL *vs*. RES neurons were conducted using IPA (Ingenuity Systems, Mountain View, CA, USA). For these analyses, all differentially expressed genes that were associated with specific biological functions in the Ingenuity Pathways Knowledge Base were considered. Fischer's exact test was used to calculate a *P *value determining the probability that each biological function assigned was due to chance alone.

For GO and Biological Pathway analyses, the lists of probesets identified to be differentially expressed between the VUL and RES groups were fed into MAPPFinder, a component of GenMAPP (Gene Map Annotator and Pathway Profiler, version 2.0) [29]. The gene and GenMAPP databases used for the analyses were those of August 2006. Hypergeometric distribution was used in MAPPFinder. A *Z *score > 1.96 or < -1.96, and a permute *P *< 0.05, were considered to be statistically significant. Gene networking analyses were performed using IPA on genes related to cell energy generation. For these analyses, the list of probesets for the genes was uploaded into IPA. Each probeset was mapped to its corresponding gene object in the Ingenuity Pathways Knowledge Base. These mapped genes were overlaid onto a global molecular network developed from information contained in the Ingenuity Pathways Knowledge Base. A gene network was then algorithmically generated based on their connectivity.

### Real-time quantitative PCR

The same RNA samples used for the microarray analyses were also used for the qPCR analyses. The RNA was first reverse-transcribed to cDNA using oligo(dT) primers and SuperScript™ II Reverse Transcriptase (Invitrogen, Carlsbad, CA, USA). TaqMan^® ^Gene Expression Assays with the following Assay IDs (Applied Biosystems, Foster City, CA, USA) were used: Rn00821759_g1 (*Mt1a*), Rn00582365_m1 (*Nefl*), Rn00582417_g1 (*Nfe2l2*), Rn00690607_m1 (*Tf*). TaqMan^® ^Fast Universal PCR Master Mix was employed for the amplification on an Applied Biosystems 7500 Fast PCR System using Fast Mode. The thermal cycling conditions were: 20 sec at 95°C (initial denaturation), followed by 40 cycles of 95°C for 3 sec and 60°C for 30 sec. β-Actin (*Actb*, TaqMan^® ^Gene Expression Assay ID Rn00667869_m1) was used as endogenous control since its expression was found to be constant across neuronal samples from the various regions. Quantification of target genes was based on the relative standard curve method [72].

## Abbreviations

**ANOVA**: analysis of variance; **BP**: biological process; **CbG**: cerebellar granular neurons; **CC**: cellular component; **CTX**: cerebral cortical neurons; **DIV**: days *in vitro*; **FCS**: fetal calf serum; **GCOS**: GeneChip Operating Software; **GenMAPP**: Gene Map Annotator and Pathway Profiler; **GO**: Gene Ontology; **GPX1**: glutathione peroxidase 1; **IPA**: Ingenuity Pathways Analysis; **LCM**: laser capture microdissection; **MF**: molecular function; **MTT**: 3-(4,5-dimethylthiazol-2-yl)-2,5-diphenyltetrazolium bromide; **OS**: oxidative stress; **PCA**: Principal Components Analysis; **PI**: propidium iodide; **qPCR**: real-time quantitative PCR; **RES**: resistant group; **RNS**: reactive nitrogen species; **ROS**: reactive oxygen species; **SNV**: selective neuronal vulnerability; **SOD1**: Cu/Zn superoxide dismutase; **SOD2**: manganese superoxide dismutase; **VUL**: vulnerable group.

## Authors' contributions

XW carried out the brain slice cultures to establish the selective neuronal vulnerability patterns observed, conducted the functional genomics studies, and drafted the manuscript. AZ carried out the cortical and cerebellar cell viability experiments and the biochemical studies on anti-oxidant proteins and ATP levels in cells in culture. RP participated in the establishment of the selective neuronal vulnerability pattern in the brain. ASG participated in the genomic studies and the real-time quantitative PCR confirmation of the GeneChip data. RB carried out the studies on the effects of different oxygen levels on the survival of primary neurons in culture. XWC participated in the bioinformatics analyses of the GeneChip data. MLM and EKM conceived of and coordinated the study, and were involved in drafting and revising the manuscript. All authors read and approved the final manuscript.

## Supplementary Material

Additional file 1 **List of currently known genes showing significantly differential expression pattern between the resistant and vulnerable groups.** Included in the list are currently known genes that showed ﹥=2 fold difference and paired t-test *P *= 0.05 between the resistant (RES) and vulnerable (VUL) groups. Uncharacterized genes (ESTs) are not included in this table. Click here for file

Additional file 2 **Network of genes related to cell energy production.** The genes highlighted in green were expressed more highly in RES as compared with VUL neurons (RES/VUL ratio shown beside each gene), whereas those not highlighted did not show differential expression levels between RES and VUL neurons. The network shown was constructed using IPA from Ingenuity. Solid lines represent direct interactions, while the broken lines indicate indirect relationships. Click here for file
