# Supplementary material for: Genomic and biochemical approaches in the discovery of mechanisms for selective neuronal vulnerability to oxidative stress
Source: BMC Neurosci. 2009 Feb 19;10:12. doi: 10.1186/1471-2202-10-12 (PMC2677396; doi:10.1186/1471-2202-10-12)
Supplement: Additional file 1 — List of currently known genes showing significantly differential expression pattern between the resistant and vulnerable groups. Included in the list are currently known genes that showed ﹥=2 fold difference and paired t-test P = 0.05 between the resistant (RES) and vulnerable (VUL) groups. Uncharacterized genes (ESTs) are not included in this table. [file 1471-2202-10-12-S1.doc]

**List of currently known genes showing significantly differential expression pattern between the resistant and vulnerable groups (≥ 2 fold difference and paired t-test *P*** ≤ 0.05)

| **Affx_ID** | **Gene Symbol** | **Description** | **Fold Difference** | **T-test *P*** | **Corrected**  ***P* **** |
| --- | --- | --- | --- | --- | --- |
| **Gene probesets showing higher expression in the resistant group (FD: RES/VUL*)** | | | | | |
| 1370810_at | *Ccnd2* | cyclin D2 | 7.67 | 6.54E-03 | 0.032 |
| 1398457_at | *Slc6a7* | solute carrier family 6 (neurotransmitter transporter, L-proline), member 7 | 7.54 | 1.33E-05 | 0.005 |
| 1389305_at | *Anxa4* | ZAP 36/annexin IV | 7.48 | 2.89E-02 | 0.067 |
| 1370419_a_at  1387957_a_at | *Sh3kbp1* | SH3-domain kinase binding protein 1 | 6.17  5.05 | 2.64E-05  3.77E-04 | 0.006  0.010 |
| 1369770_at | *Sstr1* | somatostatin receptor 1 | 6.04 | 3.66E-02 | 0.075 |
| 1373345_at | *Amigo2* | transmembrane protein AMIGO2 | 4.94 | 1.31E-02 | 0.045 |
| 1387360_at | *Stx1a* | syntaxin 1a | 4.92 | 4.55E-03 | 0.027 |
| 1369633_at | *Cxcl12* | chemokine (C-X-C motif) ligand 12 | 4.71 | 4.25E-03 | 0.026 |
| 1387914_at | *Cyp27* | cytochrome P450, family 27, subfamily a, polypeptide 1 | 4.55 | 3.87E-03 | 0.025 |
| 1369953_a_at | *Cd24* | CD24 antigen | 3.73 | 4.30E-03 | 0.027 |
| 1371094_at | *Lhx2* | LIM homeodomain protein (LH-2) mRNA sequence | 3.39 | 2.49E-04 | 0.009 |
| 1368700_at | *LOC84587* | 130kDa-Ins(1,4,5)P3 binding protein | 3.36 | 1.07E-04 | 0.008 |
| 1370058_at | *Nefl* | neurofilament, light polypeptide | 3.28 | 9.53E-04 | 0.014 |
| 1387497_at | *Npy5r* | neuropeptide Y receptor Y5 | 2.94 | 2.67E-04 | 0.009 |
| 1390358_at | *Cacna2d3* | calcium channel, voltage dependent, alpha2/delta subunit 3 | 2.91 | 1.89E-02 | 0.053 |
| 1367900_at | *Gyg* | glycogenin | 2.87 | 1.32E-02 | 0.045 |
| 1367850_at  1398246_s_at | *Fcgr3* | Fc receptor, IgG, low affinity III | 2.79  2.32 | 3.00E-03  1.59E-03 | 0.022  0.017 |
| 1368263_a_at  1370434_a_at | *Mobp* | myelin-associated oligodendrocytic basic protein | 2.76  2.42 | 1.57E-02  3.47E-02 | 0.048  0.073 |
| 1367859_at | *Tgfb3* | transforming growth factor, beta 3 | 2.69 | 1.22E-02 | 0.043 |
| 1387111_at | *Ddah1* | dimethylarginine dimethylaminohydrolase 1 | 2.68 | 2.32E-02 | 0.060 |
| 1388187_at | *Camk2a* | calcium/calmodulin-dependent protein kinase II alpha subunit | 2.63 | 2.38E-02 | 0.060 |
| 1387281_a_at | *Pnck* | pregnancy upregulated non-ubiquitously expressed CaM kinase | 2.63 | 1.46E-03 | 0.017 |
| 1376362_at | *Nptxr* | neuronal pentraxin receptor | 2.56 | 9.04E-03 | 0.038 |
| 1371007_at | *Els1* | eck-like sequence 1 | 2.55 | 1.63E-03 | 0.018 |
| 1370328_at | *Dkk3* | dickkopf homolog 3 (Xenopus laevis) | 2.55 | 6.29E-04 | 0.014 |
| 1370895_at | *Col5a2* | collagen, type V, alpha 2 | 2.54 | 1.41E-02 | 0.046 |
| 1370472_a_at | *Kcnma1* | potassium large conductance calcium-activated channel, subfamily M, alpha member 1 | 2.51 | 6.00E-03 | 0.031 |
| 1370870_at  1370067_at | *Me1* | malic enzyme 1 | 2.50  2.09 | 9.27E-03  9.11E-04 | 0.038  0.014 |
| 1370362_at | *Ptprn* | protein tyrosine phosphatase, receptor type, N | 2.46 | 6.68E-04 | 0.013 |
| 1369045_at | *Rgs14* | regulator of G-protein signaling 14 | 2.44 | 6.55E-04 | 0.013 |
| 1376191_at | *Hpgd* | NAD-dependent 15-hydroxyprostaglandin dehydrogenase | 2.43 | 3.34E-02 | 0.071 |
| 1370907_at | *Siat1* | sialyltransferase 1 | 2.40 | 2.51E-03 | 0.021 |
| 1368381_at | *W307* | W307 protein | 2.36 | 2.12E-04 | 0.010 |
| 1367652_at | *Igfbp3* | insulin-like growth factor binding protein 3 | 2.36 | 4.91E-03 | 0.029 |
| 1390196_at | *Araf1* | v-raf murine sarcoma 3611 viral oncogene homolog 1 | 2.34 | 2.14E-03 | 0.020 |
| 1370410_at | *Igsf1* | immunoglobulin superfamily, member 1 | 2.34 | 4.05E-02 | 0.080 |
| 1376051_at | *Cryl1* | crystallin, lamda 1 | 2.34 | 1.43E-03 | 0.016 |
| 1367802_at | *Sgk* | serum/glucocorticoid regulated kinase | 2.33 | 9.11E-05 | 0.009 |
| 1368810_a_at | *Mbp* | myelin basic protein | 2.33 | 1.05E-02 | 0.040 |
| 1387803_at | *Pppr2b2* | protein phosphatase 2 (formerly 2A), regulatory subunit B (PR 52), beta isoform | 2.31 | 3.82E-03 | 0.024 |
| 1368786_a_at | *Gpcr12* | G-protein coupled receptor 12 | 2.28 | 1.12E-03 | 0.015 |
| 1376711_at | *Cldn11* | claudin 11 | 2.23 | 1.15E-02 | 0.042 |
| 1387938_at | *Baalc* | brain and acute leukemia, cytoplasmic | 2.22 | 3.80E-03 | 0.025 |
| 1368417_at | *Syt5* | synaptotagmin 5 | 2.21 | 1.26E-02 | 0.043 |
| 1387836_at | *Ykt6* | prenylated SNARE protein | 2.19 | 4.31E-03 | 0.026 |
| 1370228_at | *Tf* | Transferrin | 2.17 | 2.59E-02 | 0.063 |
| 1369646_at | *Oprl* | opioid receptor-like | 2.17 | 2.16E-03 | 0.020 |
| 1367845_at | *Nef3* | neurofilament 3, medium | 2.16 | 1.19E-02 | 0.043 |
| 1369977_at | *Uchl1* | ubiquitin carboxy-terminal hydrolase L1 | 2.16 | 6.31E-03 | 0.031 |
| 1370843_at | *Gng8* | G-protein gamma 8 subunit | 2.15 | 3.00E-02 | 0.067 |
| 1371057_at | *Gabra5* | gamma-aminobutyric acid A receptor, alpha 5 | 2.14 | 1.86E-02 | 0.053 |
| 1368421_at | *Ptpn5* | protein tyrosine phosphatase, non-receptor type 5 | 2.14 | 3.76E-02 | 0.076 |
| 1368114_at | *Fgf13* | fibroblast growth factor 13 | 2.13 | 1.26E-03 | 0.016 |
| 1367586_at | *Ldha* | lactate dehydrogenase A | 2.11 | 1.24E-03 | 0.015 |
| 1387017_at | *Sqle* | squalene epoxidase | 2.10 | 2.81E-03 | 0.022 |
| 1368105_at | *Tspan2* | tetraspan 2 | 2.09 | 4.77E-02 | 0.087 |
| 1370680_at | *Stau2* | staufen, RNA binding protein, homolog 2 | 2.07 | 1.89E-03 | 0.019 |
| 1373098_at | *Band83* | band 83 | 2.07 | 1.08E-02 | 0.041 |
| 1367774_at | *Gsta1* | glutathione S-transferase, alpha 1 | 2.06 | 2.48E-02 | 0.062 |
| 1387036_at | *Hes1* | hairy and enhancer of split 1 (Drosophila) | 2.05 | 3.22E-03 | 0.023 |
| 1368247_at | *Hspa1a* | heat shock 70kD protein 1A | 2.05 | 3.98E-02 | 0.079 |
| 1387383_at | *Gpr51* | G protein-coupled receptor 51 | 2.04 | 9.89E-04 | 0.014 |
| 1370112_at | *Pten* | phosphatase and tensin homolog | 2.03 | 6.05E-03 | 0.031 |
| 1371774_at | *Sat* | spermidine/spermine N1-acetyl transferase | 2.02 | 2.93E-03 | 0.022 |
| 1374976_a_at | *Soat1* | acyl-coenzyme A:cholesterol acyltransferase | 2.01 | 1.98E-02 | 0.055 |
| 1387355_at | *Agc1* | aggrecan 1 | 2.01 | 1.63E-03 | 0.018 |
| **Gene probesets showing higher expression in the vulnerable group (FD: VUL/RES)** | | | | | |
| 1368511_at | *Bhlhb3* | basic helix-loop-helix domain containing, class B3 | 14.79 | 1.40E-02 | 0.046 |
| 1370455_a_at | *Olfm3* | olfactomedin 3 | 7.25 | 0.001173 | 0.015 |
| 1371953_at | *Ccng2* | cyclin G2 | 5.25 | 1.69E-02 | 0.050 |
| 1371039_at | *Cacnb4* | calcium channel, voltage-dependent, beta 4 subunit | 4.47 | 2.80E-02 | 0.066 |
| 1388145_at | *Tnxa* | tenascin XA | 4.22 | 1.92E-02 | 0.054 |
| 1374611_at | *Stat5b* | signal transducer and activator of transcription 5B | 4.08 | 5.84E-03 | 0.030 |
| 1367676_at | *Hmgb2* | high mobility group box 2 | 4.05 | 2.48E-02 | 0.062 |
| 1387179_at | *Adcy8* | adenylyl cyclase 8 | 3.86 | 5.06E-03 | 0.028 |
| 1370834_at | *Hs3st1* | heparan sulfate (glucosamine) 3-O-sulfotransferase 1 | 3.43 | 6.72E-03 | 0.032 |
| 1399084_at | *Dhx16* | DEAH (Asp-Glu-Ala-His) box polypeptide 16 | 3.19 | 0.037015 | 0.075 |
| 1369345_at | *Inpp4b* | inositol polyphosphate-4-phosphatase, type II, 105kD | 3.05 | 1.61E-02 | 0.049 |
| 1387476_at  1379863_at | *Kcnd2* | potassium voltage gated channel, Shal-related family, member 2 | 2.99  2.64 | 1.84E-03  8.35E-04 | 0.019  0.014 |
| 1390373_at | *Smad5* | MAD homolog 5 (Drosophila) | 2.81 | 3.78E-02 | 0.077 |
| 1386907_at | *Eno3* | enolase 3, beta | 2.74 | 1.19E-02 | 0.043 |
| 1389671_at | *Trpc2* | transient receptor potential cation channel, subfamily C, member 2 | 2.69 | 2.66E-02 | 0.064 |
| 1369200_at | *Nt5* | 5 nucleotidase | 2.66 | 6.25E-03 | 0.031 |
| 1368087_a_at | *Ptp2E* | protein tyrosine phosphatase 2E | 2.61 | 9.88E-03 | 0.040 |
| 1373268_at  1371607_at | *LOC367171* | Similar to microtubule-associated protein 4 (LOC367171), mRNA | 2.51  2.11 | 3.09E-02  1.13E-02 | 0.068  0.042 |
| 1388169_at | *LOC171120* | Pr2 protein | 2.33 | 5.04E-03 | 0.028 |
| 1370042_at | *Stmn2* | stathmin-like 2 | 2.28 | 0.046623 | 0.086 |
| 1383222_at | *LOC257646* | FERM-domain-containing protein 163SCII | 2.28 | 1.80E-02 | 0.052 |
| 1368588_at | *Rok1* | ATP-dependent, RNA helicase | 2.19 | 1.86E-02 | 0.053 |
| 1368323_at | *Tfpi* | tissue factor pathway inhibitor | 2.19 | 4.45E-02 | 0.084 |
| 1370828_at | *Zdhhc2* | zinc finger, DHHC domain containing 2 | 2.18 | 1.03E-04 | 0.009 |
| 1387397_at | *Aqp4* | aquaporin 4 | 2.16 | 0.024568 | 0.062 |
| 1376917_at | *Znf292* | Similar to zinc-finger protein (LOC313144), mRNA | 2.15 | 6.82E-03 | 0.033 |
| 1370995_at | *Pou2f1* | POU domain, class 2, transcription factor 1 | 2.09 | 1.55E-02 | 0.048 |
| 1368240_a_at | *Prkcb1* | protein kinase C, beta 1 | 2.09 | 1.33E-02 | 0.045 |
| 1371672_at | *Cbx7* | chromobox 7 | 2.05 | 3.67E-02 | 0.075 |
| 1370568_at | *Adra2c* | adrenergic receptor, alpha 2c | 2.02 | 2.98E-02 | 0.067 |
| 1390003_at | *Zfp57* | Similar to zinc finger protein 57 (LOC361783), mRNA | 2.01 | 2.76E-02 | 0.066 |
| 1389871_at | *Got2* | glutamate oxaloacetate transaminase 2 | 2.00 | 4.79E-02 | 0.087 |

* RES – Resistant Group; VUL – Vulnerable Group

** After multiple testing corrections with Benjamini and Hochberg FDR.
