# Supplementary figures and images for: Genomic and biochemical approaches in the discovery of mechanisms for selective neuronal vulnerability to oxidative stress
Source: BMC Neurosci. 2009 Feb 19;10:12. doi: 10.1186/1471-2202-10-12 (PMC2677396; doi:10.1186/1471-2202-10-12)

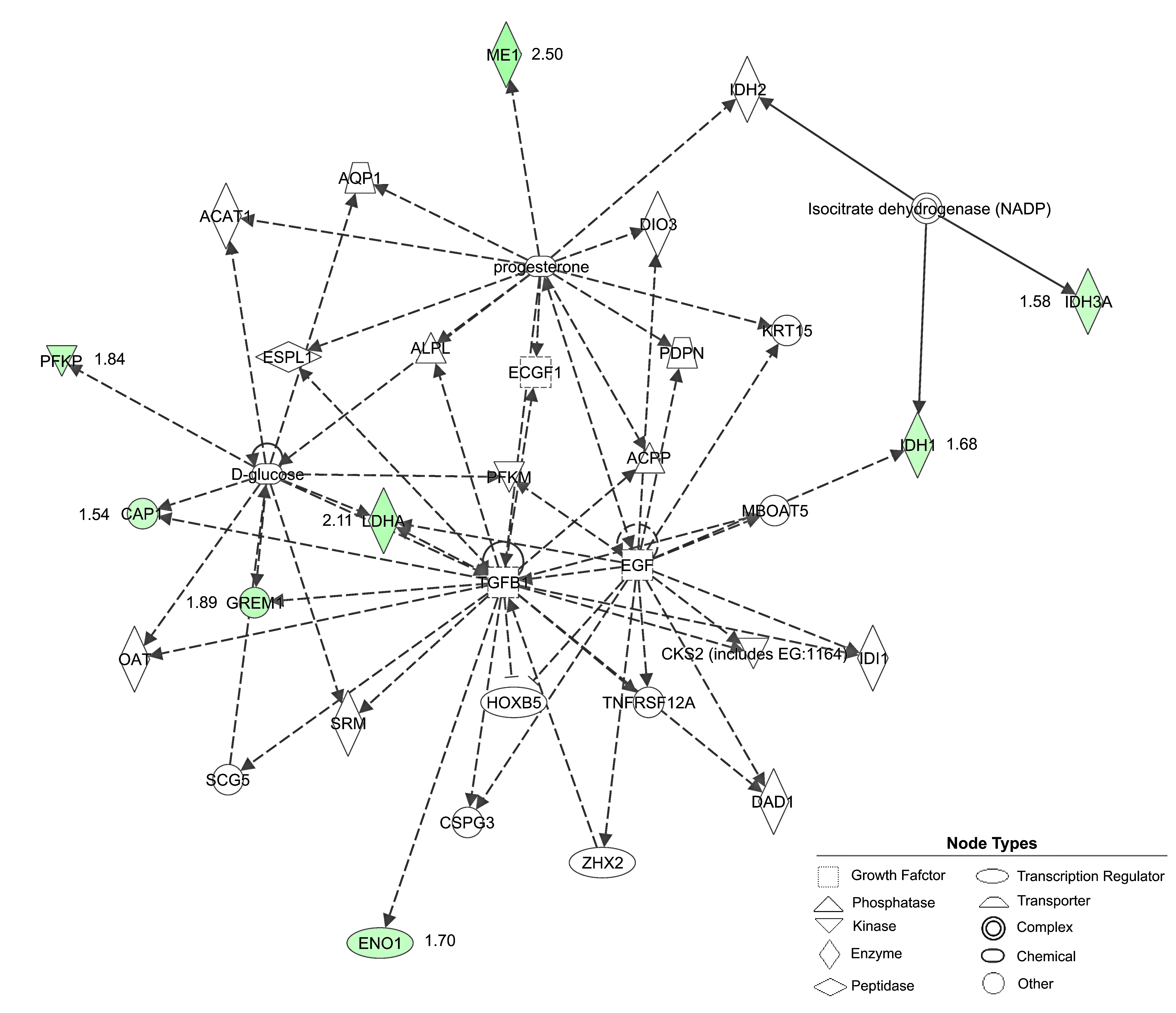

Supplement: Additional file 2 — Network of genes related to cell energy production. The genes highlighted in green were expressed more highly in RES as compared with VUL neurons (RES/VUL ratio shown beside each gene), whereas those not highlighted did not show differential expression levels between RES and VUL neurons. The network shown was constructed using IPA from Ingenuity. Solid lines represent direct interactions, while the broken lines indicate indirect relationships. [file 1471-2202-10-12-S2.tiff]
